# *In vivo* assessment of macula in eyes of healthy children 8 to 16 years old using optical coherence tomography angiography

**DOI:** 10.1038/s41598-017-08174-9

**Published:** 2017-08-21

**Authors:** Zhengwei Zhang, Xiaoli Huang, Xiaomei Meng, Tiantian Chen, Yan Gu, Yan Wu, Zhifeng Wu

**Affiliations:** Department of Ophthalmology, Nanjing Medical University Affiliated Wuxi Second Hospital, Wuxi, Jiangsu Province People’s Republic of China

## Abstract

The purpose of the present study was to investigate the vascular flow density (VD) of macular superficial (SCP), deep capillary plexus (DCP), and choriocapillaris and the size of the foveal avascular zone (FAZ) area in healthy children using optical coherence tomography angiography (OCTA). The potential associations of age, sex, intraocular pressure, body mass index, spherical equivalent, and axial length with OCTA parameters were also investigated. A total of 75 eyes from 75 healthy children were included for analysis, with the mean age 11.51 ± 1.91 years (range, 8–16 years). At the level of the SCP, mean VD and mean FAZ area were, respectively, 54.29 ± 2.25% and 0.290 ± 0.109 mm^2^. At the level of the DCP and choriocapillaris, mean VD were 60.19 ± 1.76% and 66.58 ± 1.33%, respectively. After adjustment on the signal strength index, there was no significant correlation between age and all OCTA parameters. Intra-observer repeatability was 0.91, 0.82, and 0.88 in the SCP, DCP and choriocapillaris, respectively. In healthy eyes of children, only sex has a significant influence on the FAZ area. OCTA may provide a noninvasive and reliable approach for evaluating macular perfusion in children, although sex-related variations should be considered.

## Introduction

Traditionally, fundus fluorescein angiography (FFA)^1^ and indocyanine green angiography (ICGA)^2^ are the two gold standard procedures for ophthalmic angiography. Both these procedures require injection of intravenous dye, which carries the risk of allergic reaction and is time-consuming^3, 4^. Moreover, both FFA and ICGA generate two-dimensional images of the retinal or choroidal circulation and the retinal vascular layers cannot be differentiated^5^. This poses difficulties in quantifying the perfusion of fundus vascular networks and in identifying microvascular lesions in the early stage of retinal diseases such as diabetic retinopathy.

Optical coherence tomography angiography (OCTA), a new imaging technology without the need for a dye injection, has enabled us to assess microvascular perfusion across the region of the macula and optic head disc quickly and noninvasively, offering the potential to perform quantitative assessment^6^. Two kinds of commercial equipment with different principles have been used in the living human eye^6, 7^, namely split-spectrum amplitude-decorrelation algorithm (SSADA) angiography^8^ and full-spectrum amplitude-decorrelation algorithm (FSADA) angiography^7^.

Although some new biomarkers and technologies have been used in screening childhood diabetic retinopathy^9^, OCTA may be an ideal method to avoid the complications of FFA and ICGA. Using the commercially available OCTA, many studies on healthy adults have been porformed^10–16^. Unfortunately, the use of OCTA in children is rarely reported. Thus, the normal data for OCTA in healthy children are still lacking.

In this study, we sought to report the vascular flow density (VD) of the macular superficial capillary plexus (SCP), deep capillary plexus (DCP), and choriocapillaris and the size of the foveal avascular zone (FAZ) area in healthy children *in vivo* using commercial OCTA. In addition, we analysed the influence of age, sex, body mass index (BMI), intraocular pressure (IOP), spherical equivalent refraction (SE), and axial length (AL). The findings of the present study will be helpful as a reference for children with retinal diseases, especially diabetic retinopathy.

## Results

Due to the inability to obtain images because of eye movement or blinking during image acquisition, five children were excluded. Consequently, 75 healthy children were included in the study. Both eyes of 75 healthy children were examined and successfully scanned. There were no significant differences in all associated parameters between left and right eyes (all *P* > 0.05). Subsequently, the eyes from each of the 75 healthy children with better signal strength index (SSI) were included in the present study. All study measurements provided high-quality scans, with mean SSI 73.96 ± 5.13 (range, 61 to 84).

The sample included 45 girls and 30 boys; the mean age was 11.51 ± 1.91 years (range, 8–16 years). Ocular parameters were as follows: mean IOP, 14.91 ± 2.40 mm Hg (range, 9.9–19.0 mm Hg); mean SE, −2.55 ± 2.33 diopters (range, −6.0 to 3.0 diopters); mean AL, 24.60 ± 1.07 mm (range, 20.58 to 26.97 mm); mean foveal thickness, 238.79 ± 20.53 μm (range, 178 to 307 μm). No statistically significant differences were found among the above-mentioned parameters in girls and boys (all *P* > 0.05, Table 1). The mean subfoveal choroidal thickness (SFCT) was 260.95 ± 59.34 μm (range, 156 to 435 μm), with boys (278.38 ± 59.45 μm) having greater mean values than did girls (249.32 ± 56.99 μm; *P* = 0.037). In the parafoveal region, both inner retinal and full retinal thicknesses were significant larger in boys than in girls (Table 1).

**Table 1:** Clinical characteristics of the study samples and optical coherence tomography measurements of retinal and subfoveal choroidal thickness according to sex.

	All (N = 75)	Girls (N = 40)	Boys (N = 35)	*P*
Age, years	11.51 ± 1.91	11.41 ± 1.54	11.65 ± 2.38	0.611
BMI, kg/m^2^	18.01 ± 3.10	17.66 ± 2.99	18.53 ± 3.23	0.240
IOP, mmHg	14.91 ± 2.40	14.54 ± 2.40	15.48 ± 2.32	0.094
SE, dipoters	−2.55 ± 2.33	−2.66 ± 2.41	−2.38 ± 2.23	0.605
Axial length, mm	24.60 ± 1.07	24.46 ± 1.20	24.80 ± 0.82	0.177
SSI	73.96 ± 5.13	73.87 ± 5.05	74.10 ± 5.33	0.848
**Fovea**				
full retinal thickness, μm	238.79 ± 20.53	235.22 ± 22.59	244.13 ± 15.87	0.065
SFCT, μm	260.95 ± 59.34	249.32 ± 56.99	278.38 ± 59.45	**0**.**037**
**Parafoveal region**				
inner retinal thickness^※^ μm	120.92 ± 7.69	118.56 ± 7.15	124.47 ± 7.19	**0**.**001**
full retinal thickness, μm	308.29 ± 14.30	305.04 ± 15.00	313.17 ± 11.83	**0**.**015**

^※^OCT thickness from ILM to IPL; BMI, body mess indess; SE, spherical equivalent; SSI, signal strength index; SFCT, subfoveal choroidal thickness.

The mean VD of the fovea and mean FAZ area at the level of the SCP were 31.24 ± 5.82% and 0.290 ± 0.109 mm^2^, respectively. The FAZ area was larger in girls (0.312 ± 0.114 mm^2^) than in boys (0.255 ± 0.090 mm^2^; *P* = 0.022; Table 2). However, the mean VD of the fovea was smaller in girls (29.90 ± 5.99%) than in boys (33.24 ± 4.99%; *P* = 0.014; Table 2). Apart from the two parameters, however, most of the vascular density in the SCP, DCP and choriocapillaris had no significant differences between girls and boys (all *P* > 0.05, Tables 2, 3 and 4). As for VD at the level of the choriocapillaris, there was no significant difference in the fovea and parafovea (*P* = 0.891). In the parafoveal region, the choriocapillaris had the densest vascular flow compared to DCP and SCP (*P* < 0.001).

**Table 2:** Mean vascular flow density and foveal avascular zone in the superficial capillary plexus measured by OCT angiography according to sex.

	All (N = 75)	Girls (N = 40)	Boys (N = 35)	*P*
FAZ, mm^2^	0.290 ± 0.109	0.312 ± 0.114	0.255 ± 0.090	**0**.**022**
Whole en face, %	54.29 ± 2.25	54.02 ± 2.04	54.70 ± 2.53	0.199
Fovea, %	31.24 ± 5.82	29.90 ± 5.99	33.24 ± 4.99	**0**.**014**
Parafovea, %	56.61 ± 2.60	56.45 ± 2.37	56.87 ± 2.94	0.497
Temporal, %	55.42 ± 2.33	55.04 ± 2.19	56.00 ± 2.46	0.082
Superior, %	57.60 ± 2.93	57.56 ± 2.65	57.67 ± 3.35	0.871
Nasal, %	56.18 ± 2.84	56.11 ± 2.59	56.27 ± 3.22	0.808
Inferior, %	57.22 ± 3.14	57.03 ± 3.04	57.50 ± 3.32	0.526

**Table 3:** Mean vascular flow density in the deep capillary plexus measured by OCT angiography according to sex.

	All (N = 75)	Girls (N = 40)	Boys (N = 35)	*P*
Whole en face, %	60.19 ± 1.76	60.24 ± 1.59	60.13 ± 2.01	0.788
Fovea, %	27.21 ± 6.32	26.11 ± 6.28	28.86 ± 6.10	0.064
Parafovea, %	63.66 ± 1.72	63.75 ± 1.73	63.52 ± 1.73	0.581
Temporal, %	62.32 ± 2.06	62.24 ± 2.08	62.45 ± 2.04	0.659
Superior, %	65.01 ± 2.03	64.99 ± 1.95	65.03 ± 2.17	0.938
Nasal, %	63.09 ± 1.99	63.24 ± 2.05	62.86 ± 1.90	0.425
Inferior, %	64.06 ± 2.09	64.31 ± 2.30	63.68 ± 1.97	0.222

**Table 4:** Mean vascular flow density in the choriocapillaris measured by OCT angiography according to sex.

	All (N = 75)	Girls (N = 40)	Boys (N = 35)	*P*
Whole en face, %	66.58 ± 1.33	66.68 ± 1.37	66.44 ± 1.27	0.448
Fovea, %	66.33 ± 2.31	66.31 ± 2.30	66.37 ± 2.36	0.924
Parafovea, %	66.29 ± 1.42	66.48 ± 1.48	66.00 ± 1.30	0.153
Temporal, %	66.30 ± 1.63	66.42 ± 1.66	66.11 ± 1.58	0.415
Superior, %	66.33 ± 1.57	66.64 ± 1.46	65.87 ± 1.65	**0**.**036**
Nasal, %	66.16 ± 1.80	66.28 ± 1.97	65.98 ± 1.52	0.485
Inferior, %	66.36 ± 1.63	66.58 ± 1.74	66.04 ± 1.43	0.167

**Figure 1:**
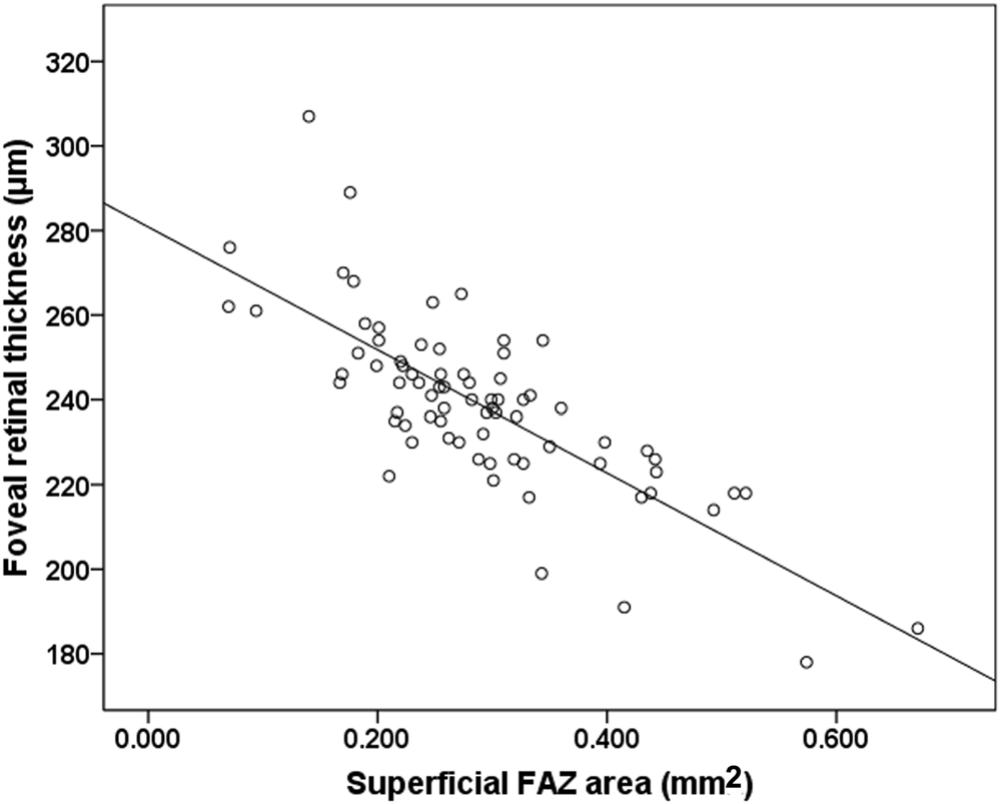
Scatterplots of association between superficial FAZ area with foveal retinal thickness. There is a strong negative correlation between the superficial FAZ area and foveal retinal thickness (*r* = −0.745, *P* < 0.001; Pearson partial analysis, adjusting for SSI).

There was a significant negative correlation between AL and SE (*r* = −0.768, *P* < 0.001; Pearson correlation analysis) or SFCT (*r* = −0.362, *P* = 0.001; Pearson correlation analysis). SSI was significantly correlated with most OCTA parameters (*P* < 0.05, Pearson partial analysis), except for the temporal (*P* = 0.057) and nasal (*P* = 0.077) vascular flow density in the DCP. Superficial FAZ area was significantly correlated with foveal retinal thickness (*r* = −0.745, *P* < 0.001; Pearson partial analysis, adjusting for SSI, Fig. 1).

In univariate linear regression analysis for all eyes (Table 5), superficial FAZ area had significant correlations with sex, axial length, foveal retinal thickness, and parafoveal retinal thickness, but did not vary significantly with age, BMI, IOP, SE, or SFCT. In multiple linear regression analysis (Table 5), superficial FAZ area varied significantly with sex, foveal retinal thickness, and parafoveal retinal thickness.

**Table 5:** Linear regression analysis of factors affecting the superficial FAZ area.

	Univariate linear regression analysis			Multiple linear regression analysis	
	Coefficient	*R* ^2^	*P*	Coefficient	*P*
**Superficial FAZ area**				*R* ^2^ = 0.771	
Sex	−0.265	0.07	**0**.**02**	−0.156	**0**.**022**
Age, years	−0.177	0.031	0.13	—	—
BMI, kg/m^2^	−0.1	0.01	0.39	—	—
IOP, mmHg	−0.016	0.0003	0.89	—	—
SE, dipoters	0.092	0.008	0.43	—	—
Axial length, mm	−0.233	0.054	**0**.**04**	—	—
Foveal retinal thickness, μm	−0.77	0.593	<**0**.**001**	−1.102	<**0**.**001**
Parafoveal retinal thickness, μm	−0.265	0.07	**0**.**02**	0.596	<**0**.**001**
SFCT, μm	−0.147	0.022	0.21	—	—
SSI	0.296	0.087	**0**.**01**	—	—

After adjusting for SSI, at the level of SCP, the foveal VD was significantly correlated with foveal retinal thickness (*r* = 0.751, *P* < 0.001); the whole en face VD was significantly correlated with parafoveal retinal thickness (*r* = 0.27, *P* = 0.02). At the level of DCP, the foveal VD was significantly correlated with foveal retinal thickness (*r* = 0.678, *P* < 0.001) and parafoveal retinal thickness (*r* = 0.23, *P* = 0.049); the whole en face VD was significantly correlated with foveal retinal thickness (*r* = 0.306, *P* = 0.008). However, there was no significant relationship between the VD of the choriocapillaris and retinal thickness in the macular region.

**Figure 2:**
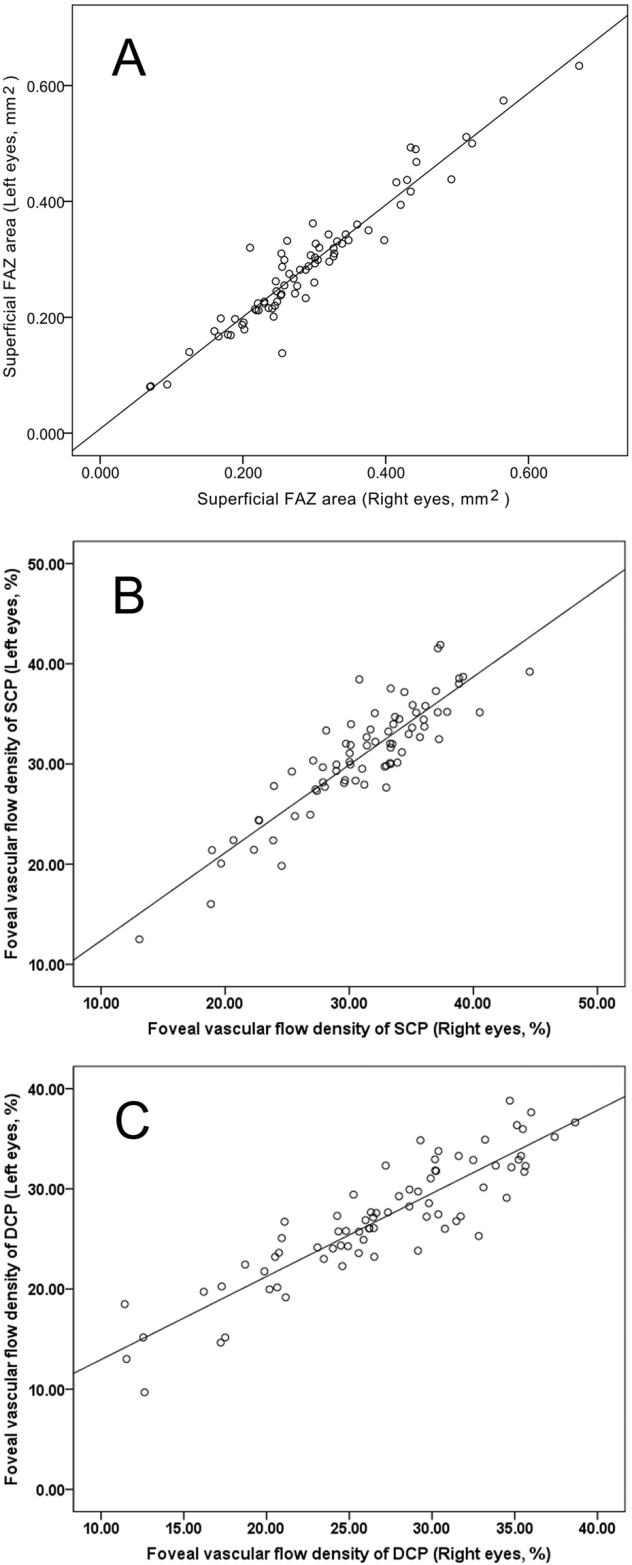
Scatterplots for FAZ (**A**), foveal vascular flow density of SCP (**B**) and DCP (**C**), showing the correlation between right and left eyes. The plots clearly show that right and left eyes are highly correlated for FAZ (*r* = 0.958, *P* < 0.001), foveal vascular flow density of SCP (*r* = 0.888, *P* < 0.001) and DCP (*r* = 0.895, *P* < 0.001).

In addition, we performed the entire analysis considering both eyes per subject, and the results were similar. As expected, there was no significant difference between the fellow eyes of our study subjects for any of the study parameters (all *P* values > 0.05). Three OCTA parameters’ correlation coefficients for bilateral eyes were more than 0.8, namely FAZ (*r* = 0.958, *P* < 0.001), foveal VD of SCP (*r* = 0.888, *P* < 0.001) and DCP (*r* = 0.895, *P* < 0.001) (Fig. 2).

For intra-observer repeatability, at the level of the SCP, the coefficient of variation (CV) and the intraclass correlation coefficient (ICC) values were 6.3% and 0.91 for the whole en face vascular flow density and 7.6% and 0.97 for the FAZ area, respectively. The CV and ICC values were 7.1% and 0.82 at the level of the DCP, and were 5.8% and 0.88 at the level of choriocapillaris for the whole en face vascular flow density, respectively (Table 6).

**Table 6:** Intraobserver repeatability of measurements of vessel density with OCT angiography in the SCP, DCP and choriocapillaris.

	FAZ		VD of the whole en face	
	ICC (95% CI)	CV, %	ICC (95% CI)	CV, %
SCP	0.97 (0.95–0.99)	7.6	0.91 (0.87–0.95)	6.3
DCP	—	—	0.82 (0.72–0.89)	7.1
Choriocapillaris	—	—	0.88 (0.80–0.94)	5.8

## Discussion

Due to the availability of the commercial equipment, OCTA is now widely used in ophthalmic clinics. Using OCTA, some studies have found that even without diabetic retinopathy, retinal VD of SCP and DCP of diabetic patients are both lower, whereas FAZ area in the superficial vascular plexus higher, compared to that in healthy subjects^17^. This indicates that retinal vascular alterations precede retinal structural alterations in diabetic patients.

Most previous studies using OCTA, however, mainly included adult patients. Consequently, very little data about children have been reported. To date, only two studies with small sample size (15 eyes as healthy controls) investigated the FAZ area and macular vessel density^18, 19^. In the present study, we investigated the VD at the level of the SCP, DCP and choriocapillaris, and the size of the FAZ at the SCP in 75 healthy Chinese children using OCTA, with the aim to add baseline OCTA data for healthy children.

Compared to the FAZ area (0.26 ± 0.09 mm^2^) of 15 eyes from 11 normal children (mean age, 7.6 ± 2.2 years) in the study conducted by Falavarjani *et al*.^18^, the FAZ area was relatively larger in our study (0.290 ± 0.109 mm^2^). The difference might derive from ethnic variation, small sample size, and subjects of relatively older age in our study. However, the FAZ area in our study was comparable to that of 15 eyes from 15 normal children (0.287 ± 0.091 mm^2^; mean age, 8.6 ± 2.2 years) in the recent study conducted by Yilmaz and associates^19^. The FAZ area of adults has been evaluated by many researchers, as summed by Shahlaee *et al*. (Table 2 in their article)^14^ and Coscas *et al*. (Table 7 in their article)^16^. Compared to the FAZ of healthy adults (mean age, 48.3 ± 17.5 years; range, 20–79 years) measured by built-in tools (0.28 ± 0.1 mm^2^)^16^, FAZ area in the present study of healthy children were relatively lager (0.290 ± 0.109 mm^2^). When considering articles that included more than 20 eyes, the FAZ area measured by OCT ranged from 0.25 mm^2^ to 0.348 mm^2^ ^20, 21^. Although the measurement methods were not identical, we could consider that the FAZ areas of healthy children are similar to those of healthy adults.

The mean FAZ area was significant larger (0.312 ± 0.114 mm^2^) in girls compared to that in boys, which were comparable to the FAZ of healthy adults (mean age, 31 ± 5 years; range, 24–41 years) measured using swept-source OCTA (0.304 ± 0.132 mm^2^)^22^. This is consistent with previous studies that reported that women had larger FAZ areas than men^23, 24^. The result from our study, for the first time, demonstrates that this sexual difference exists even in children. Of note, the effect of sex on FAZ area is still not conclusive. For example, Samara *et al*.^25^ reported that although the females did have larger FAZ areas (0.272 vs. 0.258 mm^2^) in the SCP, the difference was not statistically significant. Therefore, population-based studies are needed to determine the true effect of sex on FAZ area in the future.

In terms of FAZ area, a high inter-individual variation has been reported in normal adults. In the present study, we also found a great inter-individual variation in healthy children. In extreme cases, minimal or absent FAZ areas in healthy subjects with normal visual acuity have been reported in previous studies^11, 26, 27^, and not only in some ocular diseases^28^ or in children born preterm^18^. In our study, the FAZ areas of 6 eyes from 3 healthy children were less than 0.1 mm^2^. However, the biggest FAZ area was 0.671mm^2^. In addition, we found a significant negative correlation between foveal retinal thickness and superficial FAZ area, which was consistent with a previous study reporting the FAZ size was variable and correlated with foveal thickness in normal eyes^25^. Therefore, the foveal shape is the structural base of the size of the FAZ area. Thus, it is advisable to use one eye’s FAZ area as a reference when interpreting the other eye’s abnormality.

In multivariate analysis, superficial FAZ areas were found to be significantly influenced by sex, foveal retinal thickness, and parafoveal retinal thickness in the overall cohort (Table 5, *R* ^2^ = 0.771). However, when analysed separately, the superficial FAZ area in normal children was also affected by AL and SSI (in addition to sex, foveal retinal thickness, and parafoveal retinal thickness); the foveal retinal thickness was the most important factor to influence FAZ area (Table 5, *R* ^2^ = 0.593). This study further demonstrated that FAZ size is interrelated with foveal shape and structure.

Some previous studies reported that age had great influence in the superficial FAZ area, that is, the older the age, the smaller was the FAZ area^16^; however, some studies did not report the same findings^23, 29, 30^. All of the previous studies were cross-sectional. Thus, the real effect of age on FAZ area needed to be investigated by longitudinal studies. In the present study, the age of the included children ranged from 8 to 16 years old. This small age range cannot reveal the true picture, and no significant correlation was found between age and FAZ area as expected.

In a previous study, no significant decrease of VD in the macular region was found in high myopic eyes (SE ≤ −6.00 diopters, and without pathological changes) compared with emmetropic eyes; only macular VD decreased in pathological myopia (SE ≤ −6.00 diopters and AL ≥ 26.5 mm) compared with high myopia and emmetropia^31^. This means that there are only significant effects on the VD until pathological myopia. Consistent with this result, we also found no significant correlation between VD and refractive errors. However, there were no subjects with pathological myopia included in our study. Li *et al*.^32^ reported that the density (quantified by fractal analysis) of both superficial and deep microvascular plexuses was significantly decreased in the myopia group (−6.31 ± 1.23 diopters) in comparison to the controls (−1.40 ± 1.00 diopters). The difference might be because of the small sample size and the different method used for vascular flow density. Therefore, further studies are needed on the effect of the refractive state on VD in the macular region.

The relationship between retinal perfusion and retinal thickness in healthy subjects has been reported previously^33^. The authors found that retinal perfusion was correlated only with the inner retinal thickness (from the inner limiting membrane to the outer border of the inner nucleus layer), but not with the full foveal and parafoveal retinal thickness. In the present study, however, we found that both the full foveal and the full parafoveal thicknesses were significantly positively correlated with the foveal VD at the level of the SCP and DCP. The difference might be due to the different sample characteristics and different FAZ areas. Specifically, the mean FAZ area in our study (0.290 ± 0.109 mm^2^) was smaller than that their study (0.368 ± 0.121 mm^2^). As mentioned above, FAZ size is interrelated with foveal shape and structure. It is possible that an increase in the retinal thickness might lead to an increase in oxygen and nutrient demands, and hence increased retinal perfusion.

Many studies set a cutoff value for SSI, but did not adjust the influence of SSI when investigating correlation analyses. SSI of the OCTA scan must be considered^30^, as we found that SSI had a significant effect on the vascular flow densities and the size of FAZ, namely the higher the SSI, the greater VD and FAZ area. However, there is no consensus on what value constitutes an adequate SSI. Therefore, when evaluating the OCTA scans quantitatively, clinicians should consider the SSI value of the scan during interpretation. In the follow-up study, it is particularly essential to take SSI into account when investigating the VD and FAZ area.

Although most of the subjects in this study were young children, the reproducibility and repeatability were still high, comparable to adult subjects in previously published studies. Therefore, even young children are easy to cooperate during OCTA scanning. In our study, due to the blue fixation light and verbal encouragement, we were able to obtain sufficient signal-to-noise ratio and experienced no loss of long fixation in most of the children. Only five younger children (6.25%) failed to cooperate even after several scans.

There are a few notable limitations to the present study. First, the VD and the size of FAZ measurements evaluated in the present study were the ones automatically provided by the software. The data obtained by this means are fast and needed, because we cannot output scanned pictures to analyse for each patient in clinic. Second, the flow projection artifact of the large vessels of the SCP onto deeper retinal layers could be an issue and may have influenced the quantitative analysis of values of VD in the DCP. The newly developed projection-resolved OCTA algorithm can improve depth resolution by removing projection artifacts and help obtain accurate VD in the DCP in future studies^34, 35^. Third, any movement of the subject’s head or eyes during image acquisition would result in varying degrees of motion artifact and decreased image quality. However, Optovue’s exclusive Motion Correction Technology (MCT™) was used in the present study, which reduced the motion artifact. Fourth, because the enrolled subjects are only Chinese young children, it is possible that FAZ metrics and vascular flow values vary among different ethnicities. Last, due to abnormal vision or failure of cooperation of OCTA scans, the children younger than 8 years of age were excluded. Resultantly, the OCTA data of these children were lacking in the present study. Even with these limitations, the results from our study are noteworthy.

In conclusion, our study is the first to report that only sex has a significant influence on the FAZ area of eyes of healthy Chinese children. OCTA may provide a noninvasive and reliable approach for evaluating macular perfusion in children, although sex-related variations should be considered.

## Subjects and Methods

This was a cross-sectional study. The Institutional Review Board of Nanjing Medical University Affiliated Wuxi Second Hospital approved the protocol. All guardians of the children gave their informed consent to participate in the clinical examination program, and our study was performed in accordance with the tenets of the Declaration of Helsinki.

### Subjects

Eighty healthy children were initially recruited into this study between December 2016 and February 2017 in our ophthalmic clinic. Subjects with a history of any ophthalmic disease (except for refractive errors) were excluded. All subjects were screened for the presence of ocular diseases through a complete ophthalmologic examination, including fundus examination. Autorefraction was performed using the Topcon autorefractor (KR-8900) to obtain the refractive states of participants after cycloplegia. The mean SE was calculated as sphere power plus half the cylindrical power. The axial length was measured with the IOLMaster 500 (software version: 7.5.3.0084, Carl Zeiss Meditec, Dublin, CA). The IOLMaster is simple to use and ideal for axial length assessment, even in children^36^. To avoid upsetting the principle of independence of the observations, if both eyes met the inclusion criteria, we selected the eye with better OCTA signal strength index (SSI) from each subject for statistical analyses.

The exclusion criteria for all eyes were the following: (1) best-corrected visual acuity less than 20/20 and intraocular pressure (IOP) more than 21 mmHg; (2) refractive error (spherical equivalent) greater than +3.00 diopter or less than −6.00 diopter; (3) OCTA SSI < 60; (4) low quality of the OCTA images because of significant artifacts for poor fixation; (5) inaccurate or incorrect segmentation, or inability to obtain images from the subject because of eye movement or blinking during image acquisition; (6) poor clarity, with residual motion artifacts visible as irregular vessel pattern and local weak signal.

### OCT Acquisition and Processing

OCTA imaging was performed using the RTVue XR Avanti spectral-domain OCT device with AngioVue software (version 2016.1.0.26; Optovue, Inc., Freemont, CA, USA). This system has a light source at 840 nm and a bandwidth of 45 nm with an A-scan rate of 70 kHz. A 3 × 3-mm cube scan was acquired by two repeated B-scans at 304 raster positions, and each B-scan consisted of 304 A-scans. Two volumetric raster scans with orthogonal fast-scan directions (horizontal and vertical) were acquired for each eye and then merged to remove any motion artifact. The split-spectrum amplitude-decorrelation technology (SSADA) was applied to improve the signal-to-noise ratio^8^.

Both eyes of a subject were assessed. Each scan was automatically segmented by the AngioVue software. The SCP en face OCTA image was segmented with an inner boundary 3 μm below the internal limiting membrane (ILM) and an outer boundary set at 15 μm below the inner plexiform layer (IPL). The DCP en face OCTA image was defined as 15 and 70 μm below the IPL, respectively. All scans were reviewed to ensure correct segmentation and sufficient image quality and were repeated if deemed inadequate for analysis. Eyes with persistently low-quality scans were excluded from the study.

The VD was defined as the percentage area occupied by blood vessels, with the blood vessels defined as pixels having values above the threshold level. The AngioVue imaging system uses the proprietary SSADA algorithm to produce stunningly detailed images and to minimize scan acquisition time. Optovue’s exclusive Motion Correction Technology (MCT™) reduces motion artifact to provide ultra-high resolution images. In addition, foveal and parafoveal thicknesses were automatically calculated by the AngioVue software.

Automated measurement of the FAZ area was obtained using the nonflow area measurement option. An avascular area defined by automatic border detection was quantified. One experienced grader (Z.W.Z.) performed measurements of the FAZ area at the level of the SCP using the acquired images. The software automatically calculated the area by clicking on the centre of the FAZ. The method was described in detail elsewhere^16, 37^. Due to poorly defined borders of FAZ at the level of the DCP, it may be not accurate to measure the FAZ area automatically. Therefore, we did not include this measurement in our study analyses.

### Choroidal thickness measurement

Choroidal thickness was defined as the perpendicular distance between the outermost edge of the hyper-reflective line of the retinal pigment epithelium and the sclerochoroidal border, as reported previously^38^. Choroidal thickness was measured only under the fovea. Averaged values of the subfoveal choroidal thickness (SFCT) from the horizontal and vertical sections were recorded as the final measurement results for each eye. Two independent observers performed all measurements (Z.W.Z. and T.T.C.). Their measurements of SFCT were then averaged for statistical analysis. In case of a discrepancy in the measurement of SFCT, then the measurements were checked by another senior observer who gave the final adjudication, as reported in our previous studies^38, 39^.

### Repeatability and reproducibility

As good repeatability and reproducibility of the Optovue Avanti using SSADA algorithm have been reported in many previous studies^16, 24, 37^, OCTA possesses excellent repeatability and reproducibility indices. In the present study, we included 15 eyes to calculate the repeatability of the VD and FAZ area. The 15 eyes underwent two sets of scans performed in a single visit by the same operator. The coefficient of variation (CV) and the intraclass correlation coefficient (ICC)^40^ were calculated by comparing two measurements obtained at the same location by the same operator. The CV is the standard deviation of the measurements divided by their mean, expressed as a percentage. The ICC measures the proportion of total variability in measurements contributed by variability in measurements between different subjects. The ICC is an index of measurement reliability that ranges from 0 to 1, with values of 0.81–1.00 indicating almost perfect agreement.

### Statistical Analyses

All values are expressed as mean ± standard deviation (SD). Statistical analyses were performed using SPSS software (version 18.0, SPSS, Chicago, IL, USA). A one-sample Kolmogorov-Smirnov test was used to assess the normal distribution of continuous variables before a test of significance was applied. As a result, all of the continuous variables were normally distributed. The intersex differences were assessed using the Independent Sample *t*-test. Pearson partial correlation analysis was used to assess the relationship between OCTA and ocular parameters by adjusting SSI. Univariate and multiple linear regression analyses were performed to evaluate the relationship between superficial FAZ area and ocular factors. *P* < 0.05 was considered statistically significant. All tests were two-tailed.
